# Pneumatosis Intestinalis as the Initial Presentation of Systemic Sclerosis: A Case Report and Review of the Literature

**DOI:** 10.1155/2012/987410

**Published:** 2012-09-29

**Authors:** Farshid Ejtehadi, Nikolaos A. Chatzizacharias, Hugh Kennedy

**Affiliations:** ^1^Department of Surgery, Addenbrooke's University Hospital, Hills Road, Cambridge CB2 0QQ, UK; ^2^Department of Gastrenterology, Norfolk and Norwich University Hospital, Colney Lane, Norfolk Norwich NR4 7UY, UK

## Abstract

*Introduction*. Pneumatosis intestinalis (PI) is an uncommon pathology characterised by the presence of gas within the intestinal wall. It has been associated with various conditions, including connective tissue diseases. This is the first report of PI being the initial presentation of systemic sclerosis. *Case Presentation*. The patient, a 75-year-old female, presented with an 8-month history of worsening dysphagia and epigastric pain, as well as other nonspecific symptoms. Initial investigations with an oesophagogastroduodenoscopy diagnosed *Candida* oesophagitis and also identified an extrinsic compression of the gastric antrum. Subsequently a CT scan of the abdomen and pelvis showed moderately dilated small bowel loops and PI. Due to the patient's stability, non-critical clinical condition, conservative management was instituted. More detailed investigations confirmed the diagnosis of systemic sclerosis with positive anticentromeric and antinuclear antibodies. The patient improved on methotrexate and was discharged with appropriate outpatient follow-up. *Discussion*. PI is a rare but well-documented pathology associated with connective tissue diseases, such as systemic sclerosis. In most cases, conservative management is preferable to surgical intervention, depending on the patient's clinical presentation and progress. This is the first report of PI being the initial presentation of a patient with systemic sclerosis responsive to conservative management.

## 1. Introduction

Pneumatosis intestinalis (PI) is an uncommon pathology, first described in 1730 [1], characterised by the presence of gas within the intestinal wall, usually in the mucosa and submucosal layers of the antimesenteric border [2]. Primary PI is a benign idiopathic condition [3], while secondary PI can be associated with various underlying conditions, including necrotising enterocolitis, obstructive lung disease, inflammatory bowel disease, and connective tissue diseases [4–6]. Even though the association between PI and connective tissue diseases is well known [2, 7], antiphospholipid syndrome is the only mixed connective tissue disorder in which, it has been reported as the initial presentation [8]. To our knowledge, this is the first report of PI being the initial presentation of systemic sclerosis.

## 2. Case Presentation 

The patient was a 75-year-old female with an 8-month history of worsening dysphagia, to both solids and liquids and epigastric pain, burning in nature, radiating to the chest, costal margin, and back. Just before presentation, she experienced nausea and frequent (10–30 minutes) episodes of projectile vomiting, not related to oral intake. The patient had suffered from diarrhoea for a week prior to presentation, with no reports of blood or mucous in her stool. Generalised weakness, lethargy, reduced appetite, and a weight loss of 16 Kgs in 6 months were also reported. Past medical history was significant for hypertension and chronic obstructive pulmonary disease (COPD) under medical control.

During the clinical examination, the patient was found to have a distended, but soft abdomen. Generalised tenderness, mainly to the right side and particularly during deep palpation, was noted. No organomegaly or masses were detected. Bowel sounds were present and active. Rectal examination was unremarkable. The rest of the clinical examination only revealed bilateral pitting oedema of the ankles and swollen hands with “sausage-like” digits. 

**Figure 1:**
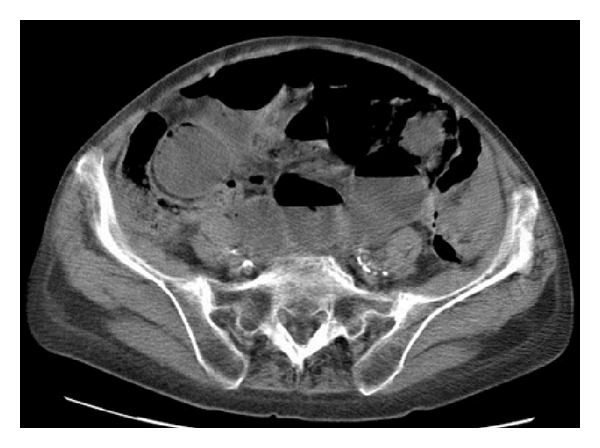
CT abdomen and pelvis showing moderately dilated small bowel loops and pneumatosis intestinalis.

The patient was initially investigated with an oesophagogastroduodenoscopy (OGD), which diagnosed *Candida *oesophagitis (possibly due to the inhaled steroids for the COPD) and mild chronic non-active inflammation of the gastric mucosa on biopsy. However, it also identified an extrinsic compression of the gastric antrum and therefore a computer tomography (CT) of the abdomen and pelvis was performed. The CT showed moderately dilated small bowel loops with gas within the intestinal wall (Figure 1). Since the patient's clinical condition was stable and did not demonstrate signs of peritonism, a decision for conservative management was made. After excluding life-threatening and acute causes of PI, such as infection or ischemia, further investigations were performed guided towards the more uncommon pathologies. A screen for autoimmune diseases showed positive results for the anticentromeric and antinuclear antibodies and the diagnosis of systemic sclerosis was made. With the diagnosis confirmed, a more detailed medical history was obtained, specifically guided to reveal any symptoms of systemic sclerosis, with no success. Similarly, no signs related to the disease, such as skin thickening, calcinosis, or telangiectasia, were identified. The patient improved on methotrexate and was discharged after a total of 4 weeks hospital stay with appropriate outpatient follow-up.

## 3. Discussion

Pneumatosis intestinalis (PI) is an uncommon pathology characterised by the presence of gas within the intestinal wall, associated with a variety of clinical disorders [2, 4, 5], including connective tissue diseases [6]. Among the latter, PI has been described in progressive systemic sclerosis, also reported as a late complication and a poor prognostic indicator [8]; systemic lupus erythematosus, where it is suggested to reflect ischemic necrosis of the bowel due to vasculitis [9]; mixed connective tissue disease [2, 7, 9–11], where it may be associated with a better prognosis [12, 13], while it is often associated with corticosteroid administration [14]. 

Although the pathophysiology of PI is uncertain, two main theories have been proposed. The mechanical theory advocates that increased luminal pressure caused by intestinal obstruction allows gas to penetrate into the submucosal space through a mucosal breach [15]. The bacterial theory supports that gas-producing bacteria, such as *Clostridium difficile *or* Clostridium perfringens*, invade the submucosal layer through mucosal rents and produce gas within the intestinal wall [16]. Finally, it has been suggested that long-term administration of corticosteroids possibly induces atrophy of the intestinal mucosa, sometimes resulting in a mucosal defect and the subsequent translocation of gas into the submucosal layer [17, 18]. This could be the explanation also in our case, where the patient was on long-term use of corticosteroids for her COPD.

The clinical importance of PI is that it may be misdiagnosed or, if identified correctly, its significance may be misinterpreted, leading to unnecessary surgical intervention [19]. Many cases of colonic gas cysts present with symptoms of large bowel dysfunction, (usually diarrhoea, colicky abdominal pain, and excessive flatulence) and, occasionally, rectal bleeding. Resection of the affected segment of the colon usually, but not always, relieves the symptoms, but in many cases spontaneous relief of symptoms can also occur [20, 21]. On the contrary, PI can also be found in cases where the viability of the bowel is threatened [6]. In these cases, it is considered to be a manifestation of a form of intestinal gas gangrene [22], and it usually indicates the need of emergency laparotomy, depending on the patient's clinical signs. In our case, the patient did not exhibit any signs of peritonism or bowel ischaemia and therefore conservative management was preferred. 

In conclusion, PI is a rare but well-documented pathology associated with connective tissue diseases, such as systemic sclerosis. In most cases, conservative management is preferable to surgical intervention, however, this decision depends on the patient's clinical presentation and progress. This is the first report of PI being the initial presentation of a patient with systemic sclerosis responsive to conservative management. 

## References

[1] Koss LG (1952). Abdominal gas cysts (pneumatosis cystoides intestinorum hominis), an analysis with a report of a case and a critical review of the literature. *A.M.A. Archives of Pathology*.

[2] Braumann C, Menenakos C, Jacobi CA (2005). Pneumatosis intestinalis—a pitfall for surgeons?. *Scandinavian Journal of Surgery*.

[3] Theisen J, Juhnke P, Stein HJ, Siewert JR (2003). Pneumatosis cystoides intestinalis coli. *Surgical Endoscopy*.

[4] Heng Y, Schuffler MD, Haggitt RC, Rohrmann CA (1995). Pneumatosis intestinalis: a review. *American Journal of Gastroenterology*.

[5] Galandiuk S, Fazio VW (1986). Pneumatosis cystoides intestinalis: a review of the literature. *Diseases of the Colon and Rectum*.

[6] Thomson WO, Gillespie G, Blumgart LH (1977). The clinical significance of pneumatosis cystoides intestinalis: a report of 5 cases. *British Journal of Surgery*.

[7] Boerner RM, Fried DB, Warshauer DM, Isaacs K (1996). Pneumatosis intestinalis. Two case reports and a retrospective review of the literature from 1985 to 1995. *Digestive Diseases and Sciences*.

[8] Fernandes C, Bungay P, O'Driscoll BR, Herrick AL (2000). Mixed connective tissue disease presenting with pneumonitis and pneumatosis intestinalis. *Arthritis & Rheumatism*.

[9] Pun YLW, Russell DM, Taggart GJ, Barraclough DRE (1991). Pneumatosis intestinalis and pneumoperitoneum complicating mixed connective tissue disease. *British Journal of Rheumatology*.

[10] Lynn JT, Gossen G, Miller A, Russell IJ (1984). Pneumatosis intestinalis in mixed connective tissue disease: two case reports and literature review. *Arthritis & Rheumatism*.

[11] Wakamatsu M, Inada K, Tsutsumi Y (1995). Mixed connective tissue disease complicated by pneumatosis cystoides intestinalis and malabsorption syndrome: case report and literature review. *Pathology International*.

[12] Van Leeuwen JCJ, Nossent JC (1992). Pneumatosis intestinalis in mixed connective tissue disease. *Netherlands Journal of Medicine*.

[13] Quiroz ES, Flannery MT, Martinez EJ, Warner EA (1995). Pneumatosis cystoides intestinalis in progressive systemic sclerosis: a case report and literature review. *American Journal of the Medical Sciences*.

[14] Vincent F, Duboust A, Glotz D (1995). Pneumatosis cystoides intestinalis and immunosuppression. *American Journal of Gastroenterology*.

[15] Keyting WS, McCarver RR, Kovarik JL, Daywitt AL (1961). Pneumatosis intestinalis: a new concept. *Radiology*.

[16] Yale CE, Balish E, Wu JP (1974). The bacterial etiology of pneumatosis cystoides intestinalis. *Archives of Surgery*.

[17] Smith BH, Welter LH (1967). Pneumatosis intestinalis. *American Journal of Clinical Pathology*.

[18] Hall RR, Anagnostou A, Kanojia M, Zander A (1984). Pneumatosis intestinalis associated with graft-versus-host disease of the intestinal tract. *Transplantation Proceedings*.

[19] Calne RY (1959). Gas cysts of the large bowel simulating multiple polyposis. *British Journal of Surgery*.

[20] Shallal JA, Van Heerden JA, Bartholomew LG, Cain JC (1974). Pneumatosis cystoides intestinalis. *Mayo Clinic Proceedings*.

[21] Sames CP (1964). Pneumatosis cystoides intestinalis. *Proceedings of the Royal Society of Medicine*.

[22] Pedersen PV, Hansen FH, Halveg AB (1976). Necrotising enterocolitis of the newborn: is it gas gangrene of the bowel?. *The Lancet*.

